# Vaccine and Non-Vaccine HPV Types Presence in Adolescents with Vertically Acquired HIV Five Years Post Gardasil Quadrivalent Vaccination: The ZIMGARD Cohort

**DOI:** 10.3390/v16010162

**Published:** 2024-01-22

**Authors:** Alltalents T. Murahwa, Tinashe Mudzviti, Racheal S. Dube Mandishora, Takudzwa Chatindo, Peace Chanetsa, Margaret Pascoe, Tinei Shamu, Wisdom Basera, Ruedi Luethy, Anna-Lise Williamson

**Affiliations:** 1 Institute of Infectious Disease and Molecular Medicine, Division of Medical Virology, Department of Pathology, University of Cape Town, Cape Town 7925, South Africa; anna-lise.williamson@uct.ac.za; 2 Wellcome Centre for Infectious Diseases Research in Africa (CIDRI-Africa), Faculty of Health Sciences, University of Cape Town, Cape Town 7925, South Africa; 3 Newlands Clinic, Harare P.O. Box A178, Zimbabwetakudzwac@newlandsclinic.org.zw (T.C.); peacec@newlandsclinic.org.zw (P.C.); margaretp@newlandsclinic.org.zw (M.P.); tineis@newlandsclinic.org.zw (T.S.); ruedi.luethy@rl-foundation.ch (R.L.); 4 Department of Pharmacy and Pharmaceutical Sciences, University of Zimbabwe, Harare P.O. Box AI78, Zimbabwe; 5 Medical Microbiology Unit, Department of Laboratory Diagnostic and Investigative Sciences, University of Zimbabwe, Harare P.O. Box AI78, Zimbabwe; racheal.mandishora@moffitt.org; 6 Center for Immunization and Infection Research in Cancer, Department of Cancer Epidemiology, Moffitt Cancer Center, Tampa, 33612 FL, USA; 7 Institute of Social and Preventive Medicine, University of Bern, 3012 Bern, Switzerland; 8 Graduate School of Health Sciences, University of Bern, 3012 Bern, Switzerland; 9 Burden of Disease Research Unit, South African Medical Research Council, Cape Town 7925, South Africa; wisdom.basera@mrc.ac.za; 10 SAMRC Gynaecological Cancer Research Centre, Faculty of Health Sciences, University of Cape Town, Cape Town 7925, South Africa

**Keywords:** human papillomavirus, Gardasil quadrivalent vaccine, human immunodeficiency virus, immunoprotection

## Abstract

Background: Human papillomavirus (HPV) vaccination programs are a key intervention in protecting individuals against HPV-related disease. HIV1-infected individuals are at increased risk of HPV-associated cancers. This study was conducted to evaluate the potential role of prophylactic HPV vaccines in preventing new HPV infections among participants with perinatally acquired HIV who received the quadrivalent HPV vaccine at least five years before this study. Methods: This cross-sectional study was conducted at Newlands Clinic, Harare, Zimbabwe. The clinic provided the Gardasil quadrivalent HPV vaccine (4vHPV) to 624 adolescents living with HIV starting in December 2015. Vaginal and penile swabs were collected and tested for HPV types from the study participants who had received the 4vHPV vaccine 5–6 years before enrolment. Results: We present the results of 98 participants (44.6% female) vaccinated at a median age of 15 years (IQR 12–16). The mean amount of time since vaccination was 6 years (SD: ±0.4). The HPV-positive rate amongst the analyzed swabs was 69% (68/98). Among 30/98 (31%) HPV-positive participants, 13/98 (13%) had low-risk HPV types, and 17/98 (17%) had high-risk HPV types. Twelve participants tested positive for HPV18, only one participant tested positive for HPV16, and an additional four (4.3%) tested positive for either type 6 or 11, with respect to vaccine-preventable low-risk HPV types. Conclusion: The Gardasil quadrivalent HPV vaccine (4vHPV) was expected to protect against infection with HPV types 16, 18, 6, and 11. We demonstrated a possible waning of immunity to HPV18 in 17% of the participants, and an associated loss in cross-protection against HPV45. We observed a relatively high prevalence of ‘opportunistic non-vaccine HPV types’ or ‘ecological niche occupiers’ in this cohort, and suggest further research on the involvement of these types in cervical and other genital cancers. Our study is one of the few, if not the first, to report on HPV vaccine immunoprotection among people living with HIV (PLWH), thereby setting a baseline for further studies on HPV vaccine effectiveness among PLWH.

## 1. Introduction

Five percent of cancers are causally associated with specific high-risk types of human papillomavirus (HPV), including cancers of the cervix, penis, vulva, vagina, anus, and oropharynx [1,2,3].

Cervical cancer is the most significant HPV-associated cancer and the leading cause of cancer-related deaths in women in developing countries. It has an estimated incidence of over 527,624, representing more than 90% of the total disease burden worldwide [4]. Mortality is considerably reduced in the developed world due to the availability of comprehensive screening and treatment of pre-cancerous and carcinoma in in situ lesions. In 2014, cancer of the cervix uteri accounted for 35.5% of all cancers occurring in women and 19% of cancer-related deaths in Zimbabwe [5]. This implies that one in every three women diagnosed with cancer had cervical cancer. There has been a call for action toward the elimination of cervical cancer as a public health problem, aiming at administering the HPV vaccine to 90% of girls under the age of 15 by 2030 [6], and a predicted reduction in the incidence of cervical cancer by 42% by 2045 [7]. Two vaccines that protect against a limited number of HPV types have been available since 2006 [8]. Another HPV vaccine has recently become available internationally. This is the Gardasil 9-valent, which protects against nine HPV types (6, 11, 16, 18, 31, 33, 45, 52, and 58) [9]. Cervarix (GlaxoSmithKline) produces antibodies to HPV types 16 and 18, while Gardasil (Merck) produces antibodies to HPV types 16, 18, 6, and 11 (the latter two types cause genital warts). Cervarix was registered in Zimbabwe in 2012. Both vaccines are sold in the private sector at a cost that is not easily affordable for the general population. Zimbabwe embarked on demonstration projects that vaccinated girls aged 9–13 years in the Marondera and Matabeleland Districts using Cervarix accessed by GAVI in early 2014 before the nationwide scale-up program in 2015–2016. The Merck nonavalent recombinant vaccine (for types 6, 11, 16, 18, 31, 33, 45, 52, and 58) is now available. The vaccine produces a much wider variety of antibodies for both HR and LR HPV types [10,11,12]. Recently, the World Health Organization (WHO) Strategic Advisory Group of Experts on Immunization (SAGE) concluded that a single-dose HPV vaccine protects against HPV in those aged 9–14 and 15–20 at a level comparable to two-dose schedules in those over 21 years old. This will remain the recommendation until there is more evidence on the impact of reduced doses in older age groups [13,14]. The importance of HPV vaccination is recognized for HIV-infected individuals, and the WHO recommends at least two, but preferably three, vaccine doses in HIV-positive individuals [14]. In people living with HIV who are vaccinated with three doses of GARDASIL, there was a decline over time in antibody titers, particularly against HPV18. A systematic review and meta-analysis showed that most studies have monitored antibodies, but because there is no known correlate of protection, the relevance of this decline is unknown [15]. However, the WHO recommends that HIV-positive individuals should receive at least two, but preferably three, doses. HPVs are part of the *Papillomavaviridae* family of viruses that mainly affect the epithelial surfaces of the skin or mucous membranes. The transmission type is ‘skin to skin’ or ‘skin to mucosal surface’. HPV is also the most common sexually transmitted infection [16]. Other transmission routes include via fomites, fingers, the mouth, and skin contact [17]. There are also some examples of perinatal transmission of HPV [18]. There is a paucity of information on HPV perinatal transmission from HIV1-positive mothers, but evidence has also been shown among HIV1-negative HPV-positive mothers [19]. As the HPV viral load is higher in HIV-positive women compared with HIV-negative women [20], it is expected that perinatal HPV transmission would be higher.

HIV infection increases the risk of persistent HPV infections and associated cancers, such as cervical and anal cancer [21,22]. Cervical cancer is an AIDS-defining illness [23], with 85% of women with AIDS in Sub-Saharan Africa being diagnosed with cervical cancer [24,25]. According to UNAIDS, approximately 39 million people globally, and an estimated 20.8 million people in East and Southern Africa, were living with HIV in 2022 [26]. Zimbabwe’s estimated population of 12 million has an HIV prevalence rate of approximately 11.6% [26]. The risk associated with increased HPV persistence and reactivation that may result from immunosuppression related to HIV is high. The risk is greater in women with low CD4+ counts (less than 200 cells/μL) and those with high plasma HIV-RNA levels [27]. HIV is associated with an increased prevalence of HPV types per subject and poor tumor differentiation [28].

This study aimed to determine if three doses of the HPV vaccine could protect against HPV infection in adolescents with vertically acquired HIV. If the vaccines are not providing protection, booster vaccination could be investigated to prevent HPV-associated disease. This would also allow policymakers to estimate the impact of the HPV vaccine and the benefits the HPV vaccination programs would achieve in the long run in HIV-positive individuals.

## 2. Methods

### 2.1. Materials and Methods

#### Study Design and Specimen Collection

This study was conducted at Newlands Clinic, Harare, Zimbabwe. Newlands is a referral clinic providing comprehensive care for PLWH. The clinic provided the quadrivalent Gardasil vaccine to 624 adolescents with perinatally acquired HIV (ALWHIV) starting in December 2015. This study was a cross-sectional study in which two study instruments were used to collect data. Firstly, the sociodemographic data were extracted from the electronic point of care (ePOC) database used at the clinic. Secondly, the data on risk factors related to this study were obtained via questionnaire interviews after seeking written informed consent from participants. The two data sets were all captured in a REDCap database for further analysis, as described below. This study was conducted between January 2021 and March 2022, and included participants vaccinated for at least five years. This study adhered to the Helsinki Declaration of 2013, and was approved by the Newlands Clinic Institutional Review Board, the Medical Research Council of Zimbabwe MRCZ/A/2668, and the University of Cape Town Ethics Committee (UCT) (HREC reference 261/2021). A total of 220 adolescent participants aged between 19 and 22 years old were recruited. The total sample size used for the reported analysis comprised 98 participants who had HPV genotyping results and complete demographic information.

### 2.2. DNA Extraction and HPV Genotyping

In this study, we collected vaginal or penile swabs in 2 mL of Digene transport media. HPV genotyping was performed using the HPV Direct Flow CHIP Kit (Vitro Master Diagnóstica, Sevilla, Spain), which isolates the DNA and qualitatively detects 35 HPV types (high-risk HPV16, -18, -26, -31, -33, -35, -39, -45, -51, -52, -53, -56, -58, -59, -66, -68, -73, and -82, and low-risk HPV6, -11, -40, -42, -43, -44, -54, -55, -61, -62, -67, -69, -70, -71, -72, -81, and -84), as described by the manufacturer. Briefly, a fragment in the viral region L1 of papillomavirus was amplified using PCR, followed by hybridization onto a membrane with DNA-specific probes using the DNA-Flow technology for manual HybriSpot platforms (Vitro Master Diagnóstica, Sevilla, Spain). The biotinylated amplicons generated after the PCR were hybridized in membranes containing an array of specific probes in a three-dimensional porous environment for each target, as well as amplification and hybridization control probes. Once the binding between the specific amplicons and their corresponding probes had occurred, the signal was visualized via an immunoenzymatic colorimetric reaction with streptavidin−phosphatase and a chromogen (NBT-BCIP) generating insoluble precipitates in the membrane in the positions featuring hybridization. The results were automatically analyzed with the HybriSoft software version 2.2.0 R00 (Vitro Master Diagnóstica, Sevilla, Spain).

### 2.3. Statistical Analyses

Questionnaire data, which included sociodemographic information, medical histories, sexual reproductive health, and participant swab results, were collected electronically via REDCap, as previously described in [29,30] with an online data-capturing web application. Statistical analyses were completed in STATA 16.0 [31] after data cleaning and exporting from REDCap. Data were presented depending on variable types, categorical data, which comprised most of the variables, and were reported as proportions or percentages, while continuous data were reported as means (±standard deviation) or medians (interquartile range) depending on the data normality. The HPV status of a participant was used as the disaggregating variable, and the statistical tests used for associations depended on the variable nature. The Mann–Whitney test or Student’s *t*-test was used to investigate differences between medians or means, respectively, according to selected categories for numerical variables. Pearson’s chi-squared test or Fisher’s exact test was used to test for associations for categorical variables. Missing data were accounted for but excluded from the data analysis on a variable-to-variable basis. A *p*-value of less than or equal to 0.05 indicated statistically significant differences or associations.

## 3. Results

Of the 220 participants recruited into the study sample, 103 had HPV genotyping results, and 91 had complete information for HPV testing and ePOC database extraction. The total sample used for the reported analysis comprised 98 participants with HPV genotyping results and most of the demographic information. The HPV-positive rate amongst the analyzed swabs was 69% (68/98). A total of 38 participants (39%) were infected with both high- and low-risk HPV types. The remaining 30 participants (31%) were either low-risk HPV DNA type positive (13% (13/98)) or high-risk HPV DNA type positive (17% (17/98)). A relatively high proportion of individuals had vaccine-type HPVs (17%), with 12 cases testing positive for HPV18, 3 for HPV6, and 1 each for HPV16 and HPV11. There were no significant differences in sociodemographic characteristics between the HPV-positive and -negative participants, except by sex (*p* = 0.01), where there were significantly more HPV-negative males than females. HPV DNA positivity was higher in older participants (21 years IQR: 20; 22) than in younger participants (20 years IQR: 19; 21) (Table 1).

**Figure 1:**
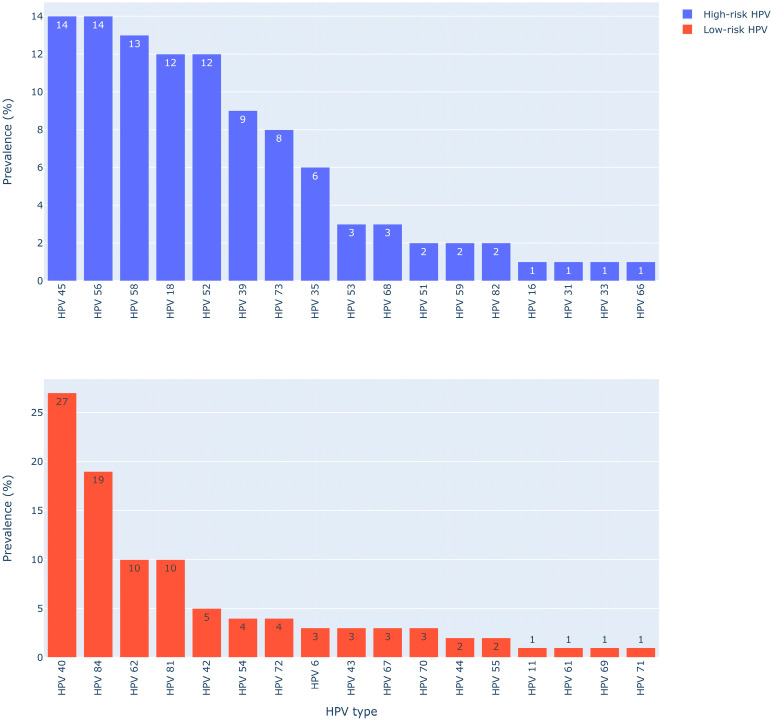
Prevalence of high- and low-risk HPV types. HPV18 was the predominant vaccine type in the cervical and penile swab samples, corresponding to 12% (12/98) of the study sample, followed by HPV6, detected in 3% (3/98) of the study samples. HPV11 and HPV16 each occurred in 1% (1/98) of all the participants (Figure 2).

Among the low-risk HPV types, HPV40 (27.6%), HPV84 (19.4%), and HPV62/81 (20%) were the most frequent (Figure 1). Among the high-risk HPV types, the most commonly detected types were HPV45 (14.3%), -56 (14.3%), -58 (13.3%), -52 (12.2%), -18 (12.2%), -39 (9.2%), -73 (8.2%), and -35 (6.19%) (Figure 1). A total of 34 of the 35 HPV types that the diagnostic genotyping test detects were detected in this cohort. Of these 34 types, only four HPV types (16, 18, 6, and 11) were vaccine types. Overall, non-vaccine HPV types had a higher prevalence than the vaccine types, such as HPV40 (27.6%) and HPV84 (19.4%). Among types expected to have cross-protection characteristics, HPV31 and HPV33 had a single case, respectively, while HPV45 had fourteen cases. Ten of the HPV45 cases occurred concurrently with HPV18 (i.e., in the same individual patients) and one with an HPV16 case. The remaining five cases occurred without any vaccine types.

A total of 17 participants (17%) had vaccine-type (VT) HPVs (HPV6, HPV11, HPV16, and HPV18) (Table 2) detected, with a higher proportion (9/17 (53%)) being male. The mean age of the VT sub-cohort was 20.7 years (±1.5), with 15 participants (88%) self-reporting being sexually active. Overall, the age of sexual debut was 19.1 years (±1.6), and there was no significant difference (ANOVA *p*-value = 0.07) when stratified by the number of sexual partners. Condoms were the contraceptive of choice in their last sexual experience and the current method of contraception used, as self-reported by ten (59%) and eight (47%) participants, respectively. A total of four participants (24%) reported having had a sexually transmitted infection (Table 2).

Generally, the mean age at vaccination among those with VT was 15.5 years (±1.6) versus 15.1 (±2.0) among those with non-VT (*p*-value = 0.67). The mean age of self-reported sexual debut among those with VT was 19.1 years (±1.6) versus 18.1 (±2.7) among those with non-VT (*p*-value = 0.27). The number of doses received by those with VT compared with the rest of the participants (i.e., those with non-VT plus HPV negativity) were one dose (1/17 (6%) vs. 0), two doses (2/17 (12%) vs. 16/79 (20%)), and three doses (14/17 (82%) vs. 63/79 (80%)) (Fisher’s exact *p*-value = 0.07).

## 4. Discussion

This is the first study conducted in Zimbabwe to determine HPV types in those vaccinated against HPV. This study targeted a population who acquired HIV via vertical transmission. They are particularly at risk for HPV-related cancers, so it is important to determine the efficacy of HPV vaccination. A key finding of this study was the significant number of HPV18 infections (*n* = 12) compared with other vaccine types (*n* = 5). We limit our discussion comments to a cross-sectional analysis of the prevalence of VTs and non-VTs in this cohort as the primary endpoint, assuming the effects of prophylactic vaccines on the prevention of HPV infection and disease have the same impact.

HPV vaccines have been established to be immunogenic in PLWH. However, further studies are necessary in the long term to evaluate the impact of vaccination and determine whether boosters are required [9]. In studies on the hepatitis B vaccine, a double dose of the HBV vaccine significantly improved the response in HIV-positive individuals. It is known for other vaccines, such as the hepatitis B vaccine, that a different dosage or immunization schedule should be used in HIV-positive individuals [32].

The Merck vaccine Gardasil and GlaxoSmithKline Cervarix for HPV have been widely reported as effective by the WHO [8] and many other studies conducted among HIV-negative cohorts [33,34]. Changes in VT HPV prevalence can be used to evaluate the vaccine impact, including the effects of herd immunity [35]. As described elsewhere, it is acceptable to use CIN2 and CIN3 and persistent HPV infection as appropriate surrogate endpoints for vaccine efficacy [36,37]. We reported a high percentage of HPV positivity (69%) among an HIV-positive cohort five years after the quadrivalent Gardasil vaccine, comprising 17 with VT HPV types and 51 with non-VTs. The VTs comprised 12 cases of HPV18, 3 cases of HPV6, and 1 each of HPV16 and HPV11.

A Scottish study that evaluated vaccine efficacy at the ages of 20–21 showed that the prevalence of HPV types 16 and 18 decreased from 30% to 4·5% after seven years of follow-up, and showed evidence of herd protection against the VT and cross-protective types in the unvaccinated individuals [33]. In an analysis of three Finnish randomized controlled cancer-registry-based follow-up studies with HPV-vaccinated groups and unvaccinated controls, HPV16/18 types were found in only three cases of CIN3 among the Gardasil and Cervarix vaccinated groups [38]. The difference between this study and the Finnish study is that the three cases were also positive for HPV16/18 at baseline, i.e., before vaccination, and so the presence of the VTs cannot be attributed to the vaccine being less effective. In our study, no baseline HPV genotyping was performed. It is well-known that HPV vaccines provide maximum benefit when administered before being sexually active [39,40,41]. A USA-based study among a group of women evaluated HPV vaccine protection nine to ten years post vaccination, and showed that VT prevalence decreased by 78% among 20–24 year-olds and 38% among 25–29 year-olds. These declines were in both vaccinated and unvaccinated women, showing evidence of direct and herd protection [35]. Most studies report the vaccine’s efficacy among HIV-negative populations compared with this study, which reported on protection among PLWH. Protection against types 16, 18, 6, and 11 with Gardasil has been reported to last for up to ten years. This was assessed in HIV-negative participants [42]. More studies are still underway to establish how long HPV vaccine protection lasts [43]. Unfortunately, most if not all the studies focus on HIV-negative cohorts in Europe and the Americas and not Africa, particularly Sub-Saharan African countries with high populations of PLWH. The dilemma of the HIV pandemic and the burden of cervical cancer in Africa warrants further investigation into HPV vaccine protection in this population.

The results also show a high prevalence of non-VT, which has been previously reported in this age group [44]. We also anticipated that after protection against the vaccine types, other opportunistic HPV types would occupy the niche created by the clearance of the vaccine-type HPVs, especially in HIV-immunocompromised individuals. A high prevalence of HPV45 (for which cross-protection is expected) was noted in our study. HPV35 has been reported elsewhere in ICC cases in South Africa [45] and Zimbabwe [46,47]. We found that cancer-causing HPV types eliminated by the vaccine were replaced by other HPV types not strongly linked to cancer, as indicated by the high prevalence of HPV52, 56, and 58 in our study. We provisionally coined these lower-oncogenicity HPV types as ´opportunistic non-vaccine HPV types’ or ‘ecological niche occupiers’. In a Finnish study on the ecological diversity profiles of non-vaccine-targeted HPVs after gender-based community vaccination efforts, there was a high prevalence of HPV52 [48], suggesting that HPV52 may be a niche occupier after the clearance of vaccine types. A Costa Rican vaccine trial demonstrated partial cross-protection with the bivalent HPV vaccine, which targets HPV16 and HPV18, against HPV31, -33, and -45 infection, and showed an increased incidence of HPV51 infection [49], also suggesting that HPV51 may be a niche occupier after the clearance of vaccine types. This warrants further investigation into all non-vaccine HPV types with a high prevalence, especially in ICC cases. It is hypothesized that vaccination can provoke ample ecological niche creation via the clearance of vaccine types. These vaccine-induced selection pressures facilitate occupation by other phylogenetically related alpha-9 species, including types 31/33/35/52/58 (besides type 16), and types 56/66 of the alpha-6 species types, which are phylogenetically close to alpha-7 species, under which HPV18 is classified [48]. The Gardasil 9 vaccine protects against types 6, 11, 16, 18, 31, 33, 45, 52, and 58, incorporating the cross-protection types into the bivalent and quadrivalent HPV vaccine.

We reported two cases and one case of type 31 and 33, respectively, which indicates the cross-protection against these types in this cohort. It has been reported elsewhere that the Gardasil quadrivalent vaccine may provide limited cross-protection against infection and lesions by HPV types 31, 33, and 45 [50,51,52]. A systematic review of the cross-protective effect of HPV vaccines reported that cross-protection is variable across non-VTs and is largely driven by HPV31 and -45 [53]. We reported 16 cases of type 45, which we assume indicates a lack of protection against this type; however, we cannot conclusively ascertain this assumption based on this study design. Ten of the HPV45 cases occurred concurrently with HPV18, and one with an HPV16 case. The remaining five cases occurred without any vaccine types. Of the 12 cases of HPV18, ten were HPV45-associated, which may suggest a loss of protection against HPV18 results in less cross-protection against HPV45. HPV18 and -45 are phylogenetically closely related, both belonging to the alpha-7 species of the *Alphpapillomaviruses* genus [54]. HPV45 is most closely related to the HPV18 African lineage based on URR sequence analyses [55]. Meanwhile, HPV31 and HPV33 are phylogenetically closely related to HPV16, belonging to the alpha-9 species of the *Alphpapillomaviruses* genus [56].

We observed that the age of vaccination was less than the age of self-reported sexual debut, meaning most of the participants were vaccinated before their first sexual encounter. HPV vaccination is most effective if received before sexual activity [39,40,41]. It is recommended to vaccinate boys and girls at the ages of 11 or 12 years [57,58]. Participants in this study were vaccinated at ages of 11 to 13 years. A study on college students between the ages of 18 and 38 also showed that sexually naïve students were more willing to receive the vaccine compared with sexually active ones [41].

A few caveats of concern regarding this study are the lack of baseline information on the HPV status of participants, the lack of an HIV-negative HPV-vaccinated control group for comparison, and the lack of a complementary surrogate marker, such as antibody titers, to corroborate the presence of the VTs. Besides these limitations, we, with reasonable validity, demonstrated the presence of VTs in this underrepresented population of ALWHIV, which was the primary endpoint.

## 5. Conclusions

We sought to determine HPV vaccine immunoprevention via HPV type surveillance in a population of HPV-vaccinated HIV-positive adolescents. We specifically focused on female and male adolescents with vertically acquired HIV infection, and concentrated on their opportunity to be protected against HPV-associated malignancies via HPV vaccination. We demonstrated a probable waning of immunity against HPV18 in 17% of the participants and an associated loss in cross-protection against HPV45. We observed a high prevalence of ‘opportunistic non-vaccine HPV types’ or ‘ecological niche occupiers’ in this cohort and suggest further research on the involvement of these types in cervical and other genital cancers. Our study is one of the few, if not the first, to report on HPV vaccine efficacy among people living with HIV (PLWH), an underrepresented population. Therefore, we have set a baseline for further studies on HPV vaccine effectiveness among PLWH.

## Figures and Tables

**Figure 2:**
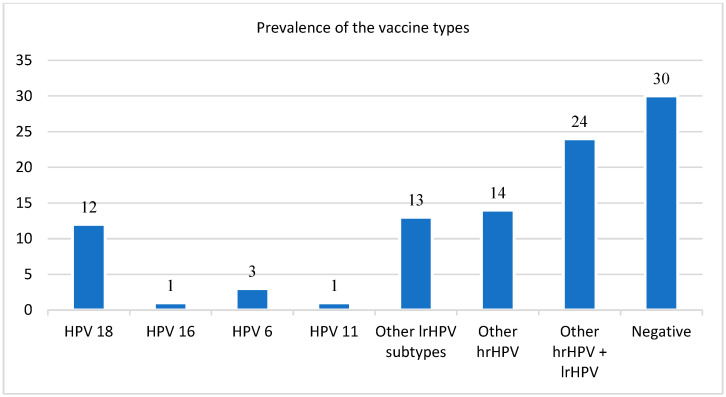
Prevalence of the vaccine-type HPVs.

**Table 1:** General sociodemographic and HPV-related characteristics disaggregated by HPV DNA test outcome.

Characteristic	Total (n_1_ = 98)	HPV− (n_2_ = 30)	HPV+ (n_3_ = 68)	*p*-Value
Sex, *n* (%)				0.01 ^
Female	42 (45)	7 (23)	35 (53)	
Male	56 (55)	23 (77)	33 (47)	
Age in years, median (IQR)	20 (19–22)	20 (19–21)	21 (20–22)	0.05 ‡
Ethnic group (Black African)	98 (95)	30 (100)	68 (93)	0.32 *
Level of education, *n* (%)				0.90 *
No schooling	3 (3)	0	3 (4)	
Primary	8 (8)	2 (7)	6 (9)	
Ordinary level	73 (74)	24 (80.0)	49 (72)	
Advanced level	13 (13)	4 (13)	9 (13)	
Tertiary	1 (1)	0	1 (1)	
First CD4+ count (cell/mm^3^) after being HIV-reactive, median (IQR) n_1_ = 55; n_2_ = 18; n_3_ = 37	320 (183–542)	402 (287–655)	280 (161–489)	0.18 ‡
Most recent CD4+ count (cell/mm^3^), median (IQR)	542 (398–731)	589 (469–782)	538 (375–714)	0.29 ‡
Nadir CD4+ count (cell/mm^3^), median (IQR)	199 (94–339)	230 (125–323)	180 (86–344)	0.38 ‡
Latest viral load copies/mL, median (IQR) n_1_ = 97; n_2_ = 30; n_3_ = 67	20 (20–33)	20 (20–21)	20 (20–39)	0.59 ‡
Latest viral load copies, *n* (%)				0.77 *
n_1_ = 97; n_2_ = 30; n_3_ = 67				
<50 copies/mL	76 (78)	24 (80)	52 (78)	
50–999 copies/mL	16 (16)	4 (13)	12 (18)	
≥1000 copies/ml	5 (5)	2 (7)	3 (4)	
Highest viral load copies/mL, median (IQR) n_1_ = 95; n_2_ = 30; n_3_ = 65	6374 (64–124, 150)	1037 (40–92, 544)	9710 (77–149, 517)	0.37 ‡
Highest viral load copies, *n* (%)				0.56 ^
n_1_ = 95; n_2_ = 30; n_3_ = 65				
<50 copies/mL	22 (23)	8 (27)	14 (22)	
50–999 copies/mL	18 (19)	7 (23)	11 (17)	
≥1000 copies/mL	55 (58)	15 (50)	40 (62)	
Time on ART (months), median (IQR)	157 (132–187)	146 (132–172)	159 (133–190)	0.31 ‡
Sociodemographic characteristics				
Current smoker, *n* (%) n_1_ = 96; n_2_ = 29; n_3_ = 67	19 (20)	4 (14)	15 (22)	0.41 *
Ever drank alcohol, *n* (%) n_1_ = 97; n_2_ = 30; n_3_ = 67	44 (45)	14 (47)	30 (45)	1.00 ^
Had sexual intercourse, *n* (%) n_1_ = 93; n_2_ = 29; n_3_ = 64	58 (62)	14 (48)	44 (69)	0.06 ^
Age at sexual debut, mean (±SD) n_1_ = 57; n_2_ = 13; n_3_ = 44	18 (±2)	18 (±2)	18 (±2)	0.59 #
Lifetime sexual partners, *n* (%)				1.00 *
n_1_ = 56; n_2_ = 13; n_3_ = 43				
One	19 (34)	4 (31)	15 (35)	
Two–four	27 (48)	7 (54)	20 (47)	
Five–nine	10 (18)	2 (15)	8 (19)	

*: Fisher’s exact test; ^: chi-square test; ‡: Mann–Whitney U test; #: Student’s *t*-test. n_1_ = total number included in analysis; n_2_ = HPV-negative number; n_3_ = HPV-positive number. *p*-value of ≤0.05 considered statistically significant. Continuous data reported as medians (IQR) or means (±SD), and *p*-values derived using the Mann–Whitney U test or Student’s *t*-test, respectively. Proportions (%) are reported as n/n_i_ (if data are missing, denominator is added in the variable name column), and *p*-values were derived using chi-squared/Fisher’s exact tests.

**Table 2:** Sexual-activity-related information of participants with isolated vaccine-type HPVs.

PID	Sex	Type	Age	Sexually Active	Age of Sexual Debut	Age at First Vaccination	Number of Vaccine Doses Received	Lifetime Number of Sexual Partners	Current Contraceptive Usage	STI History
8	Female	HPV18	22	Yes	19	17	2	2–4	None	None
17	Female	HPV18	21	Yes	21	16	3	1	None	None
26	Female	HPV18	20	Yes	19	14	3	1	Condoms	None
42	Female	HPV18	20	Yes	18	15	3	1	Condoms	Yes
50	Female	HPV18	21	Yes	17	15	3	5–9	IUD	Yes
150	Female	HPV18	23	Yes	19	18	3	1	3m injectable	None
9	Male	HPV18	22	Yes	19	17	2	2–4	Condoms	None
34	Male	HPV18	22	Yes	21	18	1	2–4	Condoms	None
58	Male	HPV18	21	Yes	19	15	3	1	IUD	None
74	Male	HPV18	18	Missing	Missing	13	3	Missing	Missing	None
75	Male	HPV18	21	Yes	15	16	3	5–9	Missing	None
90	Male	HPV18	21	Yes	21	17	3	5–9	Condoms	None
109	Female	HPV16	21	Yes	20	15	3	2–4	Condoms	Yes
64	Female	HPV6	22	Yes	20	17	3	2–4	Condoms	Yes
110	Male	HPV6	17	No	N/A	12	3	N/A	N/A	N/A
135	Male	HPV6	20	No	N/A	15	3	N/A	N/A	N/A
31	Male	HPV11	20	Yes	18	15	3	5–9	Condoms	None

## Data Availability

Data supporting the reported results can be found in the University of Cape Town REDCap database for this study, and access is available upon request from the authors.
